# The Interferon-Signature of Sjögren’s Syndrome: How Unique Biomarkers Can Identify Underlying Inflammatory and Immunopathological Mechanisms of Specific Diseases

**DOI:** 10.3389/fimmu.2013.00142

**Published:** 2013-07-05

**Authors:** Cuong Quoc Nguyen, Ammon Broughton Peck

**Affiliations:** ^1^Department of Infectious Diseases and Pathology, College of Veterinary Medicine, University of Florida, Gainesville, FL, USA; ^2^Center for Orphaned Autoimmune Diseases, University of Florida, Gainesville, FL, USA

**Keywords:** interferons, biomarkers, Sjögren syndrome, animal models, human disease, gene expression profiling

## Abstract

Innate immune responses direct the nature and specificity of downstream adaptive responses in autoimmune diseases. One of the strongest markers of innate immunity is the up-regulated expression of interferon (IFN) and IFN-responsive/stimulated genes (IRGs/ISGs). While multiple IRGs are induced during the innate phase of host responses, transcriptome data suggest unique IRG-signatures for different diseases. Sjögren’s syndrome (SjS) is characterized by chronic immune attacks against exocrine glands leading to exocrine dysfunction, plus strong up-regulated expressions of IFN IRG transcripts. Genome-wide transcriptome analyses indicate that differentially expressed IRGs are restricted during disease development and therefore define underlying etiopathological mechanisms. Here we review the innate immune-associated IFN-signature of SjS and show how differential gene expressions of IRG/ISG sets interact molecularly and biologically to identify critical details of SjS etiopathogenesis.

## Introduction

### SjS – general characteristics

#### Clinical presentation

Sjögren’s syndrome (SjS) is a chronic systemic human autoimmune disease, yet one characterized primarily by an immune-mediated reduction and destruction of lacrimal, meibomian, and salivary gland function resulting, respectively, in dry eye (keratoconjunctivitis sicca/xerophthalmia) and/or dry mouth (stomatitis sicca/xerostomia) diseases (Jonsson et al., 2000; Hansen et al., 2003; Fox, 2005; Manthorpe et al., 2006; Fox et al., 2008). However, in addition to the apparent primary sites of autoimmunity in SjS, multiple tissues can develop pathologies including the lungs, kidneys, GI tract, skin, vasculature, bladder, and vagina. Interestingly, as many as 20% of SjS patients exhibit various neuropathies, including sensory, peripheral, cranial, and myelopathic complications (Delalande et al., 2004), plus various cognitive impairments such as dementia, lack of concentration, memory loss, and various psychiatric disorders (ranging from depression to anxiety). Depression, loss of energy, and memory impairment, often noted in patients during clinic visits (Malinow et al., 1985; Belin et al., 1999; Valtysdottir et al., 2000), is referred to as “mental fogginess,” while involvement of the musculature can lead to fibromyalgia-like symptoms and chronic fatigue (Fox, 2005; Manthorpe et al., 2006). Fatigue is considered the most prevalent complaint and believed to be due to high levels of Interferon (IFN) (Iannuccelli et al., 2012). Increased IFN levels, in turn, activate multiple IFN-responsive/stimulated genes (IRGs/ISGs) involved in innate and adaptive immune activities, defining a specific SjS-associated “IFN signature.” The IFN-signature of SjS patients has been reviewed recently by Dr. Rönblom and colleagues (Nordmark et al., 2012; Yao et al., 2012).

#### SjS – the cross-over autoimmune disease

An overwhelming number of published literature supports the concept that SjS is a lymphoproliferative disorder in which B lymphocyte populations, while initiating as a polyclonal response, selectively expand temporally into monoclonal B cell populations that in about 5–10% of patients eventually transform to mucosal-associated lymphoid tissue (MALT)-associated B cell lymphomas (Isaacson and Du, 2004). In a small subset of patients, there is a gradual progression from low-grade MALT lymphomas to high-grade lymphomas (De Vita et al., 1997), thus putting the patient at risk for a life-threatening prognosis. Transformation of B cells is thought to be the consequence of constant antigenic stimulation of B cells, possibly in conjunction with the inactivation of molecular systems, like p53, and concomitant activation of bcl2 (Masaki and Sugai, 2004). While SjS is not considered a lethal disease in the absence of B cell lymphoma formation, patients have an increasingly diminished quality of life as the disease progresses (Voulgarelis and Moutsopoulos, 2003; Ansell et al., 2011).

#### Role of T and B lymphocytes in SjS

Despite the importance of B lymphocytes and autoantibodies, there is little doubt that clinical SjS is an autoimmune disease that involves both T cell and B cell participation. All autoimmune diseases appear to require activation of T cells, whether by self-antigens or environmental antigens that mimic self-antigens. However, the clinical manifestations of many autoimmune diseases, including SjS, rely on the production of autoantibodies by B cells, regulated by T lymphocytes. Nevertheless, whether T and B cells effect different clinical manifestations, at distinct times during development and onset, is still under study. Nevertheless, histology suggests that in SjS T cells tend to dominate the glandular lesions early and late while B cells tend to dominate at an intermediate phase (Nguyen et al., 2007; Nguyen and Peck, 2009). B cell development is stringently regulated by several mechanisms, including receptor editing, apoptosis, and anergy, providing many opportunities for populations of autoreactive B lymphocytes to escape tolerance-inducing mechanisms (Bemark et al., 2012). Such autoreactive B cells can become hyper-proliferative, capable of evading apoptosis, sensitive to activation, and eventually mature to produce autoantibodies (Poe et al., 2001; Niiro and Clark, 2002). In addition, hyper-proliferation of B lymphocytes contributes to an approximately 4- to 17-fold increase in the production of gamma-globulins compared with normal individuals (Pourmand et al., 1999). This polyclonal and monoclonal proliferations of autoreactive B lymphocytes lead to a state of hypergammaglobulinemia characterized by the production of organ-specific and organ-non-specific autoantibodies corresponding, for the most part, to the progression of disease development (Sawalha and Harley, 2004).

#### SjS – an IFN-signature autoimmune disease

One fascinating feature of SjS autoimmunity in both humans and animal models of SjS is the reported high levels of IFN, both IFN-α/β and IFN-γ (Hjelmervik et al., 2005; Gottenberg et al., 2006; Kawakami et al., 2007; Spachidou et al., 2007; Perez et al., 2009; Kimoto et al., 2011; Peck et al., 2011). Although elevated levels of the IFNs are often associated with viral infections, there remains little proof to date that SjS is a viral-based disease. This is despite recent observations that genes encoding TLR3, TLR7, TLR9, and factors in both the TLR- and IFN-signaling pathways are markedly up-regulated prior to the disease onset, i.e., the innate immune phase, and apparently independent of detectable adaptive autoimmunity (Wakamatsu et al., 2007; Devauchelle-Pensec et al., 2010; Obermoser and Pascual, 2010; Peck et al., 2011). Furthermore, SjS-susceptible mice expressing non-functional *Ifn*γ or *Ifn*γ*R* genes fail to develop any signs of a SjS-like disease (Perez et al., 2009; Kimoto et al., 2011), while mice expressing a non-functional *IfnaR1* gene fail to develop the clinical disease (Cha et al., 2004). Considering these observations in SjS-susceptible mouse models, the elevated levels of plasma IFNs in SjS patients and the reported activation of multiple IRGs/ISGs seen in microarray studies (Cha et al., 2002a; Dimitriou et al., 2002; Ohlsson et al., 2002; Toniato et al., 2002; Wang et al., 2008; Raterman et al., 2012), SjS, like systemic lupus erythematosus (SLE) (Yang et al., 2009) has been designated an autoimmune disease characterized by an “IFN-signature.” As stated above, this feature has been implicated as a major underlying molecular process for the high incidence of fatigue plagued patients.

## The IFN-Signature of SjS

### The SjS mouse model

Despite extensive efforts to define the genetic, environmental, and/or immunological basis for human SjS, the underlying etiology remains poorly defined. This is due, in part, to the fact that patients are currently diagnosed only after onset of overt clinical disease, sometimes as many as 10 years post-onset. In addition, patients present with multiple disease phenotypes, when considering associated pathologies beyond the three major diagnostic criteria (i.e., anti-nuclear autoantibodies, leukocytic infiltration of exocrine glands, and decreased saliva and/or tear flow rates) (Vitali et al., 2002; Shiboski et al., 2012), also remain poorly defined. In an attempt to better characterize the nature of SjS autoimmunity, an ever-increasing variety of mouse models exhibiting various aspects of SjS have been identified and studied extensively, especially as a means to investigate events associated with early-stage disease (Killedar et al., 2006; Delaleu et al., 2011). Unfortunately, the vast majority of mouse models advanced to study SjS exhibit a disease resembling more of a SLE than SjS phenotype, developing cellular infiltrates of organs but with limited evidence of concomitant sicca syndrome. Nevertheless, two mouse strains, the NOD/ShiLtJ mouse (Humphreys-Beher et al., 1994; Cha et al., 2002b) and its congenic strain C57BL/6.NOD-*Aec1Aec2* (Humphreys-Beher et al., 1994; Cha et al., 2002b), have been shown to closely mimic both the generalized SjS phenotype of humans and most of its secondary disease manifestations, as detailed elsewhere (Nguyen et al., 2007). These two mouse strains have been particularly important in studies demonstrating the importance of IFN in the pathogenesis of SjS (Cha et al., 2001, 2004) as well as defining an IFN-signature (Peck et al., 2011; Peck and Nguyen, 2012).

#### The IFN-signature of NOD/ShiLtJ and C57BL/6.NOD-*Aec1Aec2* mice

Previous publications by Cha et al. (2001, 2004) reported that high levels of IFNγ are detected in NOD/ShiLtJ and NOD-derived congenic C57BL/6.NOD-*Aec1Aec2* mice as early as the time of birth. If these SjS-susceptible mice expressed a non-functional *Ifn*γ or *Ifn*γ*r* encoding gene, they failed to develop any aspect of SjS-like disease, revealing an absolute requirement for Ifnγ in development and onset of SjS. Nevertheless, how Ifnγ plays such an important role in promoting disease in these mice remains quite speculative. In an attempt to define an IFN-signature for SjS-like disease, and one that is translatable for human SjS, follow-up studies have recently been carried out in order to analyze temporal gene-expression profiles generated for both salivary and lacrimal glands isolated from C57BL/6.NOD-*Aec1Aec2* mice for known IFN-encoding IRGs/ISGs (Peck and Nguyen, 2012). Analyses have focused heavily on genes belonging to a limited number of IRG/ISG sub-families, including *Tlr*, *Irf*, *Ifi*, *Ifr*, and *Trim* genes. The most obvious observation drawn from these analyses is the fact that only a specific subset of genes in each ISG sub-family are up-regulated, while many other genes are either neutral or down-regulated. A second observation is the fact that, of the genes whose expressions are up-regulated, one subset showed optimal expression during the innate immune stage of disease, while a second subset showed optimal expression during the adaptive immune phase of disease. Rarely, do individual IRGs/ISGs exhibit a biphasic response correlating to both the innate and adaptive immune responses. Despite these differential gene expressions, there were no direct correlations identified between the time of optimal gene expression and the expected type of IFN, but this may be due in part to the fact that multiple IRGs/ISGs are activated by both type I and type II IFN.

#### Cell-autonomous biological processes defined by the SjS IFN-signature

**Figure 1:**
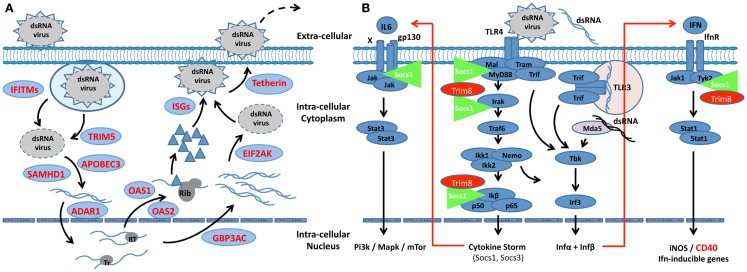
**Scheme depicting the interactive roles of interferon, Trim, and Socs molecules regulating the innate response in SjS-susceptible C57BL/6.NOD-*Aec1Aec2* mice**. **(A)** The IFN-signature observed during the early pre-clinical phase of SjS in the exocrine glands of C57BL/6.NOD-*Aec1Aec2* mice strongly suggests an autonomous cell response against a virus. Multiple IFN-responsive genes known to interfere or regulated viral replication at each step are up-regulated (shown in red). Whether this viral infection is capable of circumventing the innate response remains a viable question, as many viruses are able to regulate the innate response to their advantage, including interactions with Socs1 and Socs3. **(B)** A slowly progressing chronic infection would lead to autonomous cell responses by both membrane-associated and cytoplasmic pattern-recognition receptors (PRRs), in this case, TLR3, TLR4, and MDA-5, each initiating cellular responses via the TRIF signal transduction pathway. At the same time, signal transductions following activation of the Ifnα/β receptor involves the Jak/Tyk-Stat1/Stat2 pathway. Irf9 acts as a transcription factor that is involved in the activation of Trim molecules, many of which are E3-like ubiquitinating molecules known to interact at multiple points of viral infections, thus functioning as anti-microbial factors. Two major regulators of the INFαβ signaling pathway and the IFN-signature are Socs1, molecules that interfere with the activation loop of Jak kinase, and Trim8 that thereby preventing phosphorylation of Stat molecules. Trim8 functions as an inhibitor of Socs1, promoting continuation of IFN-signaling. Similarly, Trim21 stabilizes the function of Irf3 through blocking its interaction with Pin1, thereby promoting IFN-signaling. Genes encoding molecules that function to inhibit the innate response (Socs1, Trim27, Trim30, and Trim40) are shown to be down-regulated (green), while genes encoding factors that function to generally activate innate responses are shown to be up-regulated (red). Failure to either eliminate the etiological agent or overcome its ability to regulate the host’s innate response, most likely establishes the environment for activation of the adaptive response associated with overt clinical disease. This scheme is consistent with the strong IFN-signature observed in SjS and other rheumatic diseases, such as SLE.

The number of genes being routinely added to the *Interferome* database (Rusinova et al., 2013), together with their array of functions, underscores the fact that IRGs/ISGs are not merely activated or suppressed during development and onset of SjS-like disease, but also act as both positive and negative feedback regulatory molecules to ensure maximum host defenses against microbial infections while preventing hyperreactivity leading to unwanted host injury. Furthermore, the IFNs can no longer be viewed as purely anti-viral molecules as IFNs are a central player in innate immunity that is part of the general inflammatory response to injury. Prolonged activation of IFN signaling is critical in dealing with chronic infections, not only for activating an adaptive response, but also for orchestrating cooperative anti-microbial processes between IRGs/ISGs and autophagic factors that opsonize cytosolic pathogens or disrupt compartmentalized pathogens to facilitate efficient killing in autophagolysosomes (Macmicking, 2012). To this end, unique inducible molecular mechanisms have evolved in mammalian hosts to counter the many schemes used by microorganisms to gain entry into host cells and organs. Considering the multitude of functions displayed by IRG/ISG family proteins, one can hypothesize that global transcriptome data should distinguish between the different IFN-induced cell-autonomous effector biological processes used to kill and/or clear specific pathogens. The first consideration in such an analysis is whether the pathogen is compartmentalized, e.g., in phagocytic vacuoles or pathogen-containing inclusion bodies, or residing freely as a cytosolic pathogen. The second consideration is whether the make-up of an IFN-signature profile at the transcription level can identify, first and foremost, a specific molecular mechanism, then a specific pathogen, even though the function(s) of many IRGs/ISGs remain unknown. To date, our transcriptomic analyses strongly support the concept that the exocrine tissues are mounting an anti-viral host response and not a defensive response against bacteria or parasites (Figure 1).

#### Identification of a candidate etiological agent for SjS defined by the IFN-signature

As presented in our previous papers (Peck et al., 2011; Peck and Nguyen, 2012), analyses of global temporal transcriptome data collected during development of SjS-like disease in the C57BL/6.NOD-*Aec1Aec2* model of primary SjS defined an IFN-signature that could be used to model molecular events and their biological processes underlying SjS. Although there is little proof to date that human SjS is a viral-based disease, multiple lines of evidence clearly point to the possible role of a dsRNA viral etiology in our mouse models: (a) an up-regulated expression of *Tlr3* and *Tlr4*, two genes encoding pathogen-recognition receptors (PPRs) that signal through Traf3 via Trif and/or through Traf6 via a Trif-Trim23 complex to activate NF-κβ and Irf3/Irf7 transcription of pro-inflammatory cytokines including IFN, (b) the up-regulation of *Ifih1*, encoding Mda-5, with a concomitant down-regulation of *Ddx58*, encoding Rig-1, (c) the up-regulation of the IFN-responsive factors *Irf3*, *Irf7*, *Irf8*, and *Irf9* critical for transcription of a vast variety of genes, and (d) the down-regulation of *Trim27*, *Trim30*, and *Trim40* with concomitant up-regulation of *Trim8*, *Trim21* (encoding Ro52), *Trim25*, and *Trim56*, whose proteins impact viral replication and regulate aspects of innate immunity. While additional genes exist within each of these gene families that also exhibit differential expressions (Jefferies et al., 2011), the genes mentioned point directly to two important concepts: the first questions whether SjS might be a viral-induced autoimmunity, while the second suggests that the cytokine storm exhibited in this disease is under the direction of regulatory Trim molecules.

With respect to the first point, the three activated pathogen-recognition receptors (PRRs) in our model (Tlr3, Tlr4, and Mda-5) are receptors involved in the recognition of dsRNA viruses. We have not found any other PRR (or class of PRRs) to be activated, including Nod, Nalp, Ipaf, Naip, Rage, Rxfp1, and Dai receptors (Peck et al., 2011; Peck and Nguyen, 2012). Of particular interest, however, is the fact that *Mda5* (*Ifih1*), but not *Rig1* (*Ddx58*), is up-regulated coordinately with *Tlr3*. Rig-1 tends to recognize viruses of the *Paramyxoviridae* family (e.g., mumps, measles, respiratory syncytial and parainfluenza viruses), while Mda-5 tends to recognize viruses of the *Picornaviridae* family (e.g., coxsackie, encephalomyocarditis, and rhinoviruses) or *Reoviridae* family (e.g., rotavirus). It would be intriguing to know if SjS patients, especially those with chronic fatigue and anti-SSA/Ro and/or anti-SSB/La autoantibodies, have antibodies to viruses of these latter two virus groups.

The second point, that Trim molecules may be directing both the molecular mechanisms underlying the cytokine storm observed in SjS patients and the transition from an enhanced innate response to an adaptive autoimmune response, is strongly supported by the *Trim* gene-expression profile present in the exocrine glands (Jefferies et al., 2011). In essence, the three Trim molecules (Trim27, Trim30, and Trim40), whose gene expressions are down-regulated, function to suppress the signal transductions of the Tlr4, Tlr3, and Mda5 signaling pathways at various signaling points. In contrast, the genes encoding Trim21, Trim23, Trim25, and Trim56, four molecules whose functions are to up-regulate the Tlr3, Tlr4, and Mda5 pathways at different signaling steps, are each up-regulated. In addition, the gene encoding *Trim8*, whose function is to suppress the action of the Socs (Suppressor of cytokine synthesis) molecules (Toniato et al., 2002) is strongly up-regulated. Taken as a whole, this profile indicates up-regulation of pathways leading to strong transcription of pro-inflammatory cytokines, IFNs and molecules known to activate adaptive responses (e.g., IL6, IL12p40, Rantes, CD40, CD80, and CD56). Not surprising, then, is that the innate phase of SjS transitions to an adaptive immune phase, but this data still raises a question regarding whether or not viruses, known to have strong interactions with Trim molecules, are responsible for the temporal differential gene-expression profiles observed at the transcriptome level.

#### Comparison between mouse and human SjS-associated IFN-signatures

One encouraging aspect of transcriptome data thus far published for SjS, although still limited, is the fact that genes used to establish the IFN-signatures in both mouse and humans overlap (Table 1). This is true even though the specific underlying etiologic agents are suspected to be different. An additional confounding issue is that each inbred mouse model represents a single genetic background; whereas the human disease is heterogeneous both genetically and phenotypically. Furthermore, the disease time-points being analyzed are clearly not the same. Nevertheless, the IRGs/ISGs that have thus far been reported as differentially expressed in human SjS patients by several groups (Hjelmervik et al., 2005; Gottenberg et al., 2006; Wakamatsu et al., 2007; Emamian et al., 2009; Perez et al., 2009; Devauchelle-Pensec et al., 2010; Kimoto et al., 2011) include *IRF7, MX1, GIP2, GIP3, OAS1, OAS2, PKR, IFI16, IFI27, IFI30, IFI35, IFI44, ISG20, ISG56K, IFIT1, IFIT2, IFIT4, IFITM1, IFITM3*, *IP10/CXCL10, APOBEC3, SAMHD1, TETHERIN, VIPERIN*, and *STAT1a*. The majority of these are also represented in the differentially expressed genes up-regulated in the exocrine glands of the C57BL/6.NOD-*Aec1Aec2* mice (Peck and Nguyen, 2012). At the same time, the numbers of IRGs/ISGs up-regulated and differentially expressed in SjS patients most likely represent only a fraction of the total possible IRGs/ISGs. Thus, this overlapping set of differentially expressed genes must be considered an important subset of responsive genes that we would hypothesize point to specific etiopathological processes.

**Table 1:** **Comparison between mouse and human SjS-associated IFN-signature genes**.

Gene family	Mouse	Human
IFN-induced GTPase	Igtp, ligp1	
IFR	ifrg15	
IFIT	Ifit1, Ifit3	IFIT1, IFIT2, IFIT4
IFITM	Ifitm2, Ifitm3	IFITM1, IFITM3
IRF	Irf1, Irf3, Irf6, Irf7, Irf8, Irf9	IRF7
ISG/ISGF	Isg20/1	ISGF-3 (STAT1a), ISG20, ISG56K
IFI/IFIH	Ifi35, Ifi47, Ifi202b, Ifi205, Ifih1	IFI4 (OAS1), OAS2, IFI10 (IP10/CXCL10), IFI16, IFI27, IFI30, IFI35, IFI44, IFI-78K (MX1), VIPERIN, SAMHD1
Antiretroviral defense		BST-2 (TETHERIN), APOBE

Despite the fact that a significant number of SjS patients, if tested during clinic visits, can present with elevated levels of plasma IFN, the human transcriptome data indicate that few, if any, genes encoding an IFN *per se* exhibited up-regulation as compared to normal, healthy individuals (Hjelmervik et al., 2005; Emamian et al., 2009). In contrast, transcriptome data indicate that at the same time multiple IRGs/ISGs are up-regulated, a fact published first by Hjelmervik et al. (2005) using human minor salivary glands (huMSGs), then by Emamian et al. (2009) using peripheral blood mononuclear cells (PBMCs), and replicated in the exocrine glands of SjS^S^ C57BL/6.NOD-*Aec1Aec2* mice. This observation suggests that, in the starting salivary gland tissue, there may be limited numbers of plasmacytoid dendritic cells (pDCs), which are a major source of IFNs. Although it can be argued that these data result from the fact that IFN quickly binds to their target receptors, we have interpreted these results to suggest that the earliest disease stage(s) actually occur(s) outside of the targeted exocrine glands where pDCs are likely to occur and in higher numbers, and/or the type 1 IFN expression highly relevant for innate immunity is rapidly replaced by the type 2 IFN expression strongly associated with adaptive immunity. Alternatively, if the etiological agent does turn out to be a virus, then one cannot rule out the possibility that the source of IFN in SjS, besides pDCs, is the autonomous innate response by the glandular epithelium *per se* involving Ifnα5 (Peck et al., 2011; Peck and Nguyen, 2012).

While it is natural to focus on the many similarities in the IFN-associated gene sets differentially expressed in human SjS patients and SjS-susceptible C57BL/6.NOD-*Aec1Aec2* mice, there are also important major differences. A few that stand out include expression profiles for *Mx1*, *Irf8*, *Ifi202b – Ifi205* encoding the p200 family molecules, and the three IFN-inducible genes, *Ifi27*, *Ifi30*, and *Ifi44*. One might expect a difference in *Mx1* expression, as laboratory mouse strains, especially C57BL/6J, which are thought to carry a non-coding *Mx1* gene (Staeheli and Sutcliffe, 1988). On the other hand, the *Irf8* gene, which encodes a factor that is involved in myeloid differentiation and Fas-mediated apoptosis as well as B cell development and transcriptional regulation of germinal center formation (Wang and Morse, 2009), deserves special attention due to its highly up-regulated expression in the C57BL/6.NOD-*Aec1Aec2* mice. Our earlier studies postulated that myeloid cells enter the exocrine glands during the early innate response (8–12 weeks of age) in response to Fas-FasL-mediated apoptosis of acinar tissue, while B cells enter the salivary glands transiently during the adaptive immune phase (post-16 weeks of age). Interestingly, the temporal expression profile of *Irf8* showed a bimodal profile in line with this hypothesis. The inability to detect an up-regulated expression of *IRF8* in SjS patients is an interesting aspect to examine further. This is because binding of the transcriptional factor PU.1 to Irf8 leads to up-regulation of OAS1 and/or OAS2, two molecules that can bind and degrade dsRNA viral RNA (Rogozin et al., 2003), and are highly up-regulated in SjS patients. In contrast, the p200 molecules, encoded by the *Ifi200* family of genes, are known to sense cytoplasmic DNA, leading to the formation and activation of inflammasomes with subsequent production of anti-nuclear antibodies (Choubey et al., 2010). Although there was an *Ifi202b* up-regulated gene expression in the exocrine glands of C57BL/6.NOD-*Aec1Aec2* mice, we have not found evidence for activation of inflammasomes in these mice, based on gene expressions of PRRs, in contrast to their comparative SjS-non-susceptible C57BL/6J partner (Peck and Nguyen, 2012). Lastly, whereas *IFI27*, *IFI30*, and *IFI44* have been consistently found to be up-regulated in SjS patients (Hjelmervik et al., 2005; Emamian et al., 2009; Devauchelle-Pensec et al., 2010; Kimoto et al., 2011), these three *Ifi* genes with distinct functions were not found to be differentially expressed in the exocrine glands of C57BL/6.NOD-*Aec1Aec2* mice. Considering IFI44 is associated with HCV and RSV infections, we would contend that this difference between humans and mice lies in the fact that the underlying etiological agent(s) of SjS in these two species is different and invokes different environmental triggers. Interestingly, IFI44L was identified as a marker gene in RA (Raterman et al., 2012). Thus, differentially expressed genes common to both species probably indicate activation of similar pathways of immunopathological processes more important than individual genes. This highlights the fact that both similarities and differences in the IFN-signatures will be critical to understanding SjS.

## Perspectives

Despite efforts to define an environmental, genetic, and/or immunopathological basis for SjS, the underlying etiology remains poorly defined with little consensus in the field. This is, in part, due to the fact that patients are currently diagnosed only after the onset of overt clinical disease, and then showing the presence of multiple disease phenotypes when considering associated pathologies beyond the three major diagnostic criteria. However, transcriptome studies that are beginning to define a “disease-specific IFN-signature profile” appear to offer a viable approach, if not an absolute answer, for developing hypotheses for further testing. To this end, we believe that the IFN-signature of the C57BL/6.NOD-*Aec1Aec2* mouse model points directly to a cytoplasmic dsRNA viral etiology and a dysregulated innate immune response giving rise to an autoimmune inflammatory pathology. Support for these concepts lies in the observations that: (a) the three activated PRRs in our model (Tlr3, Tlr4, and Mda-5) are receptors involved in activating the IFN-based innate response against dsRNA viruses; (b) the genes associated with cell-autonomous immune effector mechanisms exhibiting up-regulated expressions generally defines an anti-cytoplasmic viral response; and (c) the expression of specific Trim and Socs molecules known to regulate the IFN pathway remain in a balance favorable for activating, not down-regulating, innate immunity. Taken as a whole, this overall IFN profile indicates up-regulation of pathways leading to strong transcription of IFNs, pro-inflammatory cytokines, and molecules that are known activators of adaptive responses (e.g., IL6, IL12p40, Rantes, CD40, CD80, and CD56). The result is a prolonged innate phase of SjS that favors transitions to an adaptive immune phase rather than resolution.

The unique temporal changes exhibited by IFN-responsive genes involved in molecular and biological processes reveal differential expressions of selected subsets of genes. Detection of which differentially expressed genes are crucial to specific molecular processes and which genes are merely normal responses remains complicated. Any measurement at one time point of disease development is a serious weakness of applying transcriptome data analysis to human autoimmune diseases; but no doubt, represents an incomplete picture. This still defines critical elements of the etiopathological processes. Studies using the C57BL/6.NOD-*Aec1Aec2* mouse model of primary SjS document the fact that there are multiple IRGs/ISGs that are not differentially expressed, and that this lack of gene-expression is not due to disease phase-restricted expressions. This observation, therefore, invokes a need to consider both up-regulated and down-regulated genes in defining a disease-specific IFN-signature. In conclusion, there is a need to determine both the similarities and differences in IFN-signatures between diseases within a single species in order to establish how an environmental trigger might circumvent naturally built-in mechanisms that are in place to prevent diseases, and if a specific IFN-signature points to the underlying etiological agent.

## General Comments

Considering the significant number of IRGs/ISGs, together with the biological pathways regulated by these genes, one should not be surprised that a disease such as SjS demonstrates a restricted and, most probably, a unique transcriptomic profile. At the same time, the specific genes that are observed to be up-regulated, plus those that are either silent or down-regulated, appear to identify molecular pathways and biological processes that point to a specific etiology, and possibly the etiological agent underlying disease *per se*. While much of the data discussed in the current review is based on our own temporal transcriptome analyses of C57BL/6.NOD-*Aec1Aec2* mice, there are multiple similarities with SjS in human patients, despite the contention that the two diseases, most likely, are not caused by identical etiological agents. For this reason, it is important for temporal transcriptome studies to be carried out, as best possible, in SjS patients to determine exactly how similar they actually are. At the same time, genomic transcriptome studies need to be conducted in other diseases with a diverse etiology in order to compare the IFN-signatures. At this time, we would predict that each disease will exhibit a unique IFN-signature, especially during the innate phase of disease, and that the IFN-signature would be diagnostic. As such, intervention into the individual disease would start sooner and be more specific.

## Conflict of Interest Statement

The authors declare that the research was conducted in the absence of any commercial or financial relationships that could be construed as a potential conflict of interest.
